# Home and Office Treatment of Inebriety

**Published:** 1907-03

**Authors:** 


### HOME AND OFFICE TREATMENT OF INEBRIETY.

Dr. T. D. Crothers says that efforts to give temporary relief are noted by the following examples: A prominent lawyer who was to be a candidate in a coming convention applied to a physician for help to prevent him from drinking. He was given large doses of quassia for several days before the convention, with pills of atropia and strychnine at night. He passed through this period without the least impulse to drink, and later continued the treatment and permanently recovered. Another case was that of a half-crazed, trembling inebriate calling at the office of a physician and in a most pathetic way describing the impulses he had to kill his wife and child and commit suicide, thus ending the suffering. He had called on a number of physicians who had all turned him away in disgust, some giving him money, others morphia and spirits. The physician gave him a large dose of apomorphine, and after free vomiting gave him a bed in his stable. This was repeated during the day and night, and the next day he was able to go home. The physician continued the treatment and followed the case up regularly for several months until full recovery followed. These are by no means unusual or startling instances. The possibility of their occurrence is not unusual in the practice of every physician. At present thoughtless physicians give placebos, morphia, chloral and other narcotics to these office patients and not infrequently they are found dead a few hours later.

The time is not far distant when every medical man of the country will recognize the gravity of acute intoxication and treat such persons successfully. Where the treatment cannot be carried out at home, hospitals and sanitariums will assist in removing the acuteness of the conditions and securing a degree of restoration which can be followed up by the family physician at home.—Va. Medical Semi-Monthly, December 7th, 1907.

